# Evaluating the Implementation of a Before-School Physical Activity Program: A Mixed-Methods Approach in Massachusetts, 2018

**DOI:** 10.5888/pcd17.190445

**Published:** 2020-10-01

**Authors:** Rachel C. Whooten, Christine Horan, Jack Cordes, Anna Nicole Dartley, Annabelle Aguirre, Elsie M. Taveras

**Affiliations:** 1 Division of General Academic Pediatrics, Department of Pediatrics, Massachusetts General Hospital for Children, Boston, Massachusetts; 2 Division of Endocrinology, Department of Pediatrics, Massachusetts General Hospital for Children, Boston, Massachusetts; 3 Department of Epidemiology, Harvard TH Chan School of Public Health, Boston, Massachusetts; 4 Kraft Center for Community Health, Massachusetts General Hospital, Boston, Massachusetts; 5 Department of Nutrition, Harvard TH Chan School of Public Health, Boston, Massachusetts

## Abstract

**Purpose and Objectives:**

Our aim was to evaluate the implementation of a widely available, before-school, physical activity program in a low-resource, racially/ethnically and socioeconomically diverse, urban school setting to identify adaptations needed for successful implementation.

**Intervention Approach:**

We used a collaborative effort with stakeholders to implement the Build Our Kids’ Success (BOKS) program in 3 schools in Revere, Massachusetts. Program structure followed a preexisting curriculum, including 60-minute sessions, 3 mornings per week, over 2 sessions (spring and fall 2018). Programs had a capacity of 40 students per school per session and the ability to adapt as needed.

**Evaluation Methods:**

We used a mixed-methods approach, guided by the Reach, Effectiveness, Adoption, Implementation, and Maintenance (RE-AIM) framework. RE-AIM domains were assessed by use of baseline and follow-up student measures, parent interviews, and program administrative records.

**Results:**

From a district of 11 schools, 3 schools (2 elementary, 1 middle) implemented the BOKS program. Program enrollment reached 82% capacity (188 of 230 potential participants). Of the 188 enrolled students, 128 (68%) had parental consent for study participation. Among the 128 study participants, 61 (48%) were male, 52 (41%) identified as Hispanic/Latino, and mean age was 9.3 years (SD, 2.2). Program duration varied by school (25–60 minutes), with a mean of 33% (SD, 16%) of the session spent in actigraphy-measured moderate-to-vigorous physical activity (MVPA), or mean 16.3 (SD, 9.3) minutes of MVPA. Participants attended a median 90% (interquartile range [IQR], 56%–97%) of sessions. We observed no change in body mass index (BMI) *z* score or self-reported quality of life from baseline to follow-up assessment. Parents reported positive program effects. Enrollment was sustained in elementary schools and decreased in the middle school during the study period, expanding to 3 additional schools for spring 2019.

**Implications for Public Health:**

Implementation and evaluation of an evidence-based physical activity program, in a low-resource setting, are feasible and yield relevant information about program adaptations and future dissemination of similar programs.

**Summary**

**What is already known on this topic?**

Despite strong evidence supporting school-based physical activity interventions, a knowledge gap exists that relates to intervention delivery in real-world settings.

**What is added by this report?**

We evaluated the implementation of a before-school physical activity program in 3 schools by using a structured evaluation framework. Each school had different approaches to program delivery, with potential implications for program results and sustainability.

**What are the implications for public health practice?**

Use of a structured implementation evaluation framework provides key insights into program implementation and delivery that can be used to guide future dissemination efforts across different settings.

## Introduction

Physical activity is an important lifestyle behavior that is associated with a reduced risk of chronic diseases (1) and is known to have other benefits, ranging from improved cognition (2) to social and emotional wellness (3,4). Data from 2018 indicate that most children do not meet recommendations for 60 minutes of physical activity daily (5); therefore, increasing physical activity levels is an important population health target for interventions. The school setting has been proposed as an effective place to reach children across socioeconomic levels without barriers that might exist in other community-based settings (6).

Despite strong evidence supporting a role for school-based physical activity interventions (7), a significant knowledge gap exists related to intervention delivery in real-world settings (8). Most studies focus on intervention effectiveness, with limited reporting on program implementation (9). Public health results depend on successfully disseminating and diffusing effective interventions (10). To adequately address dissemination and diffusion, we must understand how interventions are adopted, implemented, and sustained in less controlled settings, especially relative to the need for adaptations (11).

Use of the Reach, Effectiveness, Adoption, Implementation, and Maintenance (RE-AIM) framework allows investigators to systematically address the gap between research and practice, recognizing that optimal research conditions often do not reflect the complexity of real-world settings (12). By understanding initial intervention adoption and reach, followed by implementation, effectiveness, and maintenance (Table 1), the RE-AIM framework seeks to capture both internal and external validity and facilitate the translation of research findings into real-world settings (12).

**Table 1:** Assessment Measures in the RE-AIM Framework^a ^of the Build Our Kids’ Success Program (BOKS) Evaluation, Massachusetts, 2018

RE-AIM Dimension	Definition	Source of Data (Level)	Data Collected	Tools
**Adoption**	Support and uptake for adoption of programming	School	School demographics Trainer recruitment	District records Administrative data
**Reach**	Proportion of target population participating in intervention	School	Number of students eligible	District records
			Number of students enrolled	Enrollment records
		Child	Participant characteristics	Enrollment records Anthropometrics
		Parent	Parent feedback	Semi-structured interviews
**Implementation**	Extent to which intervention is implemented as intended in the real world	Program	Program structure	Administrative records
			Program content	SOFIT structured observation
			Physical activity delivery	Accelerometry
			Program costs	Administrative records
			Logistical support	Stakeholder conversations
		Child	Program attendance	Enrollment records
		Parent	Parent feedback	Semi-structured interviews
**Effectiveness**	Success if implemented as intended	Child	BMI (*z* score)	Anthropometrics (height, weight)
			Quality of life	Pediatric Quality of Life Inventory
		Parent	Parent feedback	Semi-structured interviews
**Maintenance**	Extent to which program is sustained over time	School	Number of students enrolled in fall 2018 vs spring 2018	Enrollment records
			Trainer retention	Administrative data
			Program attendance	Enrollment records

Abbreviations: BMI, body mass index; RE-AIM, Reach, Effectiveness, Adoption, Implementation, and Maintenance; SOFIT, System for Observing Fitness Instruction Time.

a Based on Glasgow, et al (12).

Our study used a mixed-methods design, guided by the RE-AIM framework, to evaluate an evidence-based, before-school physical activity program, Build Our Kids’ Success, or the BOKS program, in 3 public schools in a low-income community in Revere, Massachusetts. Previous studies showed the effectiveness of BOKS in improving child weight status, social–emotional wellness, and overall physical activity levels (13,14); however, less is known about program delivery and outcomes in low-resource settings. We hypothesized that a detailed implementation evaluation would identify adaptations to program structure, with potential implications for program outcomes.

## Purpose and Objectives

The primary goal of this study was to perform a structured implementation evaluation of a widely available and preexisting before-school physical activity program, Build Our Kids’ Success (BOKS), in a low-resource setting and to identify adaptation needs and targets for optimizing future interventions in similar settings. The BOKS program includes a freely available curriculum, designed for delivery by trained volunteers, with sessions occurring 2 to 3 times weekly during a 12-week period. For our study, the BOKS program was implemented and evaluated for 2 sessions of 12 weeks each (March–May 2018, October–December 2018) in collaboration with stakeholders, including community health infrastructure, and school district administration.

The BOKS program aligns with a systems-level framework of obesity, which highlights the importance of considering both the interpersonal and community levels in effective obesity prevention initiatives (15). The RE-AIM evaluation framework (Table 1) is consistent with this approach by facilitating understanding of intervention contexts (8,12).

## Intervention Approach

Implementation of BOKS was supported by a Massachusetts General Hospital community health improvement grant awarded to target health disparities in Revere, Massachusetts. In this community, 65% of students speak a first language other than English, 47% are from economically disadvantaged families, defined as participation in ≥1 state-administered program, including public health insurance, food assistance, or child protective services (16), and 66% identify as a racial/ethnic minority, primarily Hispanic/Latino (17). The city has a high burden of chronic disease, with 47% of children meeting the criteria for overweight/obesity (18); rates of diabetes, stroke, and cardiovascular disease among adults are higher than the state average (19).

**Figure 1:**
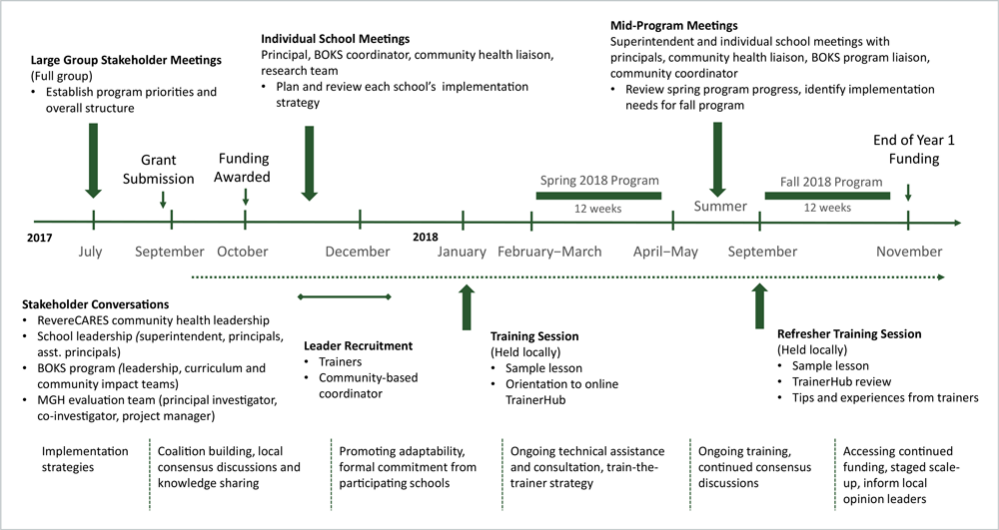
Evaluation timeline with overview of stakeholder engagement process and implementation strategies, 2018–2019.

Before the initiative was funded, our research team participated in collaborative community engagement meetings, forming a coalition with local community health leaders, school principals, and BOKS program staff to align program and evaluation priorities. This group met throughout the project to discuss program implementation and delivery (Figure 1).

For our study, 3 schools committed to implementing the BOKS program for 1 year, beginning in the spring of 2018. School leadership identified candidate schools in the target community and selected 1 middle school (grades 6–8) and 2 elementary schools (grades K–5) from 11 schools in the district. Each participating school recruited 2 trainers to lead the program, enrolled students, and completed 2 sessions of the BOKS program for 12 weeks, 1 session in spring 2018 (February–June) and 1 session in fall 2018 (September–December).

Because BOKS is widely available, the primary intention was to deliver the program within the standardized BOKS curriculum; however, we prioritized each school’s ability to adapt the program as needed to suit their delivery capabilities. A grant-funded, school-based coordinator assisted each school, working in consultation with a coordinator from the BOKS program. We chose this train-the-trainer approach to build capacity in the community.

Before the start dates of the spring and fall programs, program leaders from each school attended a 2-hour BOKS training session held in the community. Training consisted of an introduction to the online TrainerHub at https://www.bokskids.org/, which provided resources and access to the standardized curriculum as well as participation in a sample lesson. Each school had the capacity for 40 students per 12-week session, based on the BOKS standard of 1 trainer for every 20 students. Total program capacity was originally 120 maximum students enrolled per session (240 students, over 2 sessions of 12 weeks, across 3 schools). Maximum capacity decreased to 230 students following one school’s decision to limit capacity to 30 students for their fall program.

## Evaluation Methods

We performed an exploratory concurrent-nested mixed-methods evaluation, guided by the RE-AIM framework. We chose this approach to embed supportive qualitative data within the larger quantitative evaluation (20). We collected evaluation data at the individual level (student, parent) and at program and school levels (Table 1).

The study was conducted from February 2018 through December 2018 and included 2 BOKS program sessions with program maintenance observed through spring 2019. The study was recorded in clinicaltrials.gov (NCT#2017P002770) and approved by the Partners HealthCare Institutional Review Board of Boston, Massachusetts.

All children enrolled in the 3 participating schools (K–8) were eligible to enroll in the BOKS program, although 1 school excluded kindergarten because of scheduling conflicts. Each school directed program enrollment at the start of the spring and fall sessions. To increase program access for the fall session, schools gave enrollment preference to children who had not previously participated.

To facilitate enrollment, study staff provided schools with information and recruitment materials to send home with children at the start of each session. Materials were available in English, Spanish, Arabic, and Portuguese. Parents returned signed consent forms in a sealed envelope to the school, where study staff picked them up. A telephone number was included for parents to call and ask questions. For collection of child measures, children provided verbal assent to trained research staff at the start of study visits.

**Figure 2:**
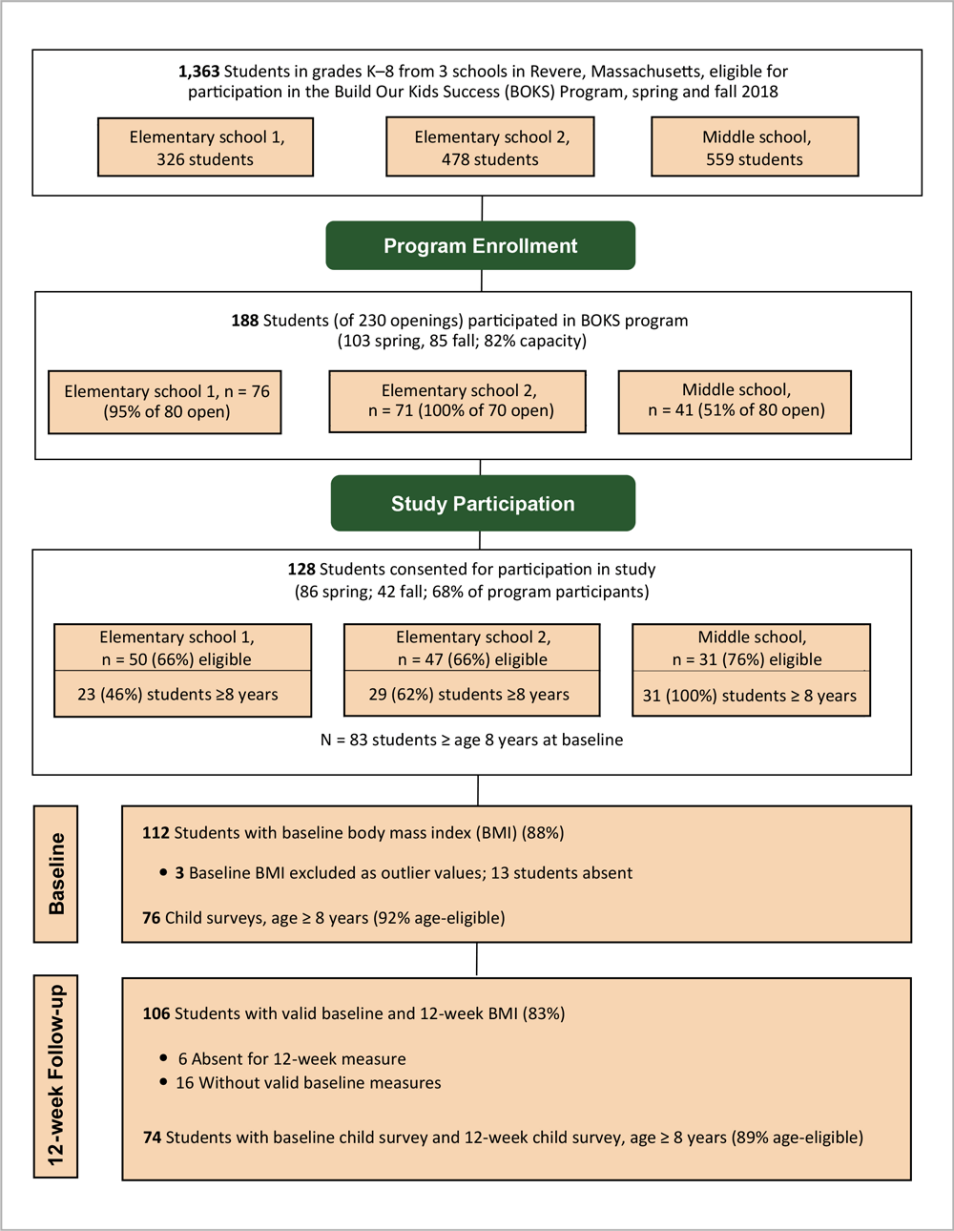
Flow diagram of physical activity participants in the Build our Kids Success (BOKS) evaluation from 3 schools in Revere, Massachusetts, spring and fall 2018.

Students were eligible to participate in the study with enrollment in the BOKS program, valid written parental consent, and verbal child assent. Of 188 total children enrolled in the BOKS program during the study period, 128 (68%) had both parental consent and child assent for study participation (Figure 2). Although children whose parents did not consent to study enrollment could continue their participation within the BOKS program, we did not collect any study measures from them other than anonymized attendance.

Parents from 1 participating elementary school formed a convenience sample for a 20-minute semistructured, qualitative telephone interview. Parents received a letter describing the interview and returned a form providing permission to be contacted. Eligibility criteria included being an English-speaking parent and having a child enrolled in the BOKS program. Parents provided verbal consent at the start of the interview. We randomly selected participants from interested parents until the target sample size was reached (n = 20). Ultimately, 25 of 40 parents (62.5%) expressed interest, 23 were contacted for participation, 20 completed the interview, and 2 were not contacted because the target sample size was already reached. Parents received a $10 gift certificate when they completed the interview. Interviews were conducted by trained research assistants and were recorded and transcribed for qualitative analysis.

For evaluating program adoption, school records provided information on district and school demographics (17). We compared demographics of participating schools to overall district demographics. Program administrative records provided information on trainer recruitment. District records detailed the number of students eligible for participation in each school. We obtained program enrollment data from registration forms and attendance records to evaluate program reach. Baseline participant characteristics included race/ethnicity, sex, and age (obtained from registration forms) as well as anthropometrics (obtained through study visits). We compared participant demographics at baseline to overall school demographics. Parents also answered semistructured interview questions addressing reasons for enrolling their child in the BOKS program.

Implementation evaluation occurred at both the program and individual levels. Program administrative records provided information on program costs. We assessed intervention fidelity through review of program structure, administrative records, and session visits. Trained research assistants visited each school at least once (range 1–3 times) per 12-week session to perform a structured physical activity observation and obtain objective measurement of participant physical activity.

Research staff conducted structured observations by using the System for Observing Fitness Instruction Time (SOFIT) (21,22). With this tool, independent observers classified student activity and lesson context in 10-second intervals. Classifications for activity included vigorous activity, walking, standing, sitting, and lying down. Classifications for lesson context included fitness, games, skills, knowledge, and management. Frequencies of each activity and context classification were calculated, and percentage of each classification within total number of observations was reported. Session observations with interobserver reliability of 75% or less were excluded (n = 2). We performed a total of 10 structured observations using SOFIT, with 87% interobserver reliability for activity observations and 84% for context observations.

On session observation days, 10 to 15 study participants wore an ActigraphGT3X+ (Actigraph, LLC) accelerometer on the wrist or an ActigraphGTX on the hip. Upon arrival to the morning program, research staff fitted children with the accelerometer and recorded time for program arrival (monitor on) and program departure (monitor off). We recorded physical activity intensity levels using Evenson cut-point thresholds (23). Children were included for analysis if total wear time was consistent with program duration. We made objective measurements of physical activity during 9 sessions and obtained 84 total observations.

Individual-level data included child attendance and parent feedback through semistructured qualitative interviews on program feasibility and acceptability, including barriers to program participation and parental input on program content and structure. Stakeholder conversations throughout the intervention planning and implementation periods documented logistical support provided for programming.

Effectiveness evaluation included individual child measures, as well as qualitative interview data from parents on observed program effectiveness among children. Research staff collected anthropometrics and quality-of-life data at baseline and 12-week follow-up during both sessions (spring and fall). Trained research assistants measured child weight and height using a Seca scale and stadiometer (Seca North America East Medical Scales & Measuring Devices). We calculated child body mass index (BMI) and age- and sex-specific BMI *z* score for each participant (24). Additionally, students aged 8 years or older completed the Pediatric Quality of Life Inventory 4.0 (PedsQL) Child Self Report, a reliable and valid measure of health-related quality of life in a healthy population (25). The PedsQL consists of 23 items addressing physical, emotional, school, and social domains. This measure was self-administered on paper. For program maintenance, study staff reviewed program and administrative records for fall session enrollment, attendance, and trainer retention and for spring 2019 enrollment.

### Data analysis

We tabulated descriptive statistics of participant characteristics by session and school. We assessed differences between sessions and schools by using *t* tests, ANOVA, and Wilcoxon tests as appropriate for continuous variables, and χ^2 ^for categorical variables. We assessed all data for outlier values and errors in data entry.

For the analyses of anthropometrics and quality of life, we used linear regression to measure change across assessment points, using each assessment point as a categorical predictor and model coefficients to estimate change in outcome from baseline. We included all available cases for analyses, with only complete cases included in measurement of change from baseline to each assessment point. For objective measurement of physical activity, we calculated means for wear time as well as time spent in MVPA. We performed 1-way ANOVA to evaluate differences between schools and frequencies for participants achieving thresholds of MVPA. We also report a Pearson correlation coefficient to describe correlation between program duration and MVPA minutes. Due to nonnormality of attendance, we report median and interquartile ranges. We performed Wilcoxon tests to assess for difference in attendance by school, spring versus fall program participation, identification as Hispanic/Latino, and study participant versus nonparticipants.

At maximum program capacity (n = 230), this study would be powered to detect a 0.38 unit change in BMI *z* score, with 80% power at significance level *P* = 0.05. However, with actual enrollment at 188, we were underpowered to detect statistically significant change in BMI *z* score so we report the observed difference from baseline to follow up only. We performed quantitative analyses using R 3.5.0 (R Core Team) (26). We collected and managed study data using REDCap electronic data capture tools hosted at Partners Healthcare (27).

All interviews were recorded and transcribed for analyses. We conducted thematic analyses using the Framework Approach (28), establishing an a priori deductive framework within relevant RE-AIM domains (29). Two coders independently reviewed transcripts, developed preliminary themes and codes, and compared initial framework to reach consensus. Coders then indexed themes and relevant quotations into an Excel spreadsheet from line-by-line transcript review, refining by combining and removing codes as needed to generate thematic framework. Coders resolved any discrepancies through discussions.

## Results

### Adoption

Grant funding for implementation of the BOKS program was available for 3 schools in a district with 11 schools. Overall, participating schools were representative of the district in the percentage of economically disadvantaged students (district 47.1%; participating school range 45.4%–51.0%) and students whose first language was not English (district 64.5%; participating schools range 61.7%–65.8%) (17).

A total of 11 program trainers were trained across all sessions and schools. Elementary school 1 had 2 trainers, with the addition of an occasional parent volunteer. Elementary school 2 recruited 6 trainers in total, with 2 present each morning. The middle school recruited 3 trainers; 1 led both sessions and the other 2 led 1 session each. Roles of trainers in the school included gym teacher (n = 2), school nurse (n = 1), academic teacher (n = 7), and counselor (n = 1); 9 of 11 trainers were female. One trainer served as the grant-funded community coordinator and was responsible for coordination within schools and primary communication with the BOKS program.

### Reach

At maximum capacity, the program could accommodate 230 students over 2 sessions, or approximately 17% of the total student body across schools. A total of 188 students (82%) of potentially 230 students began the program. Total enrollment for the fall session was reduced to 110 students, as 1 school (elementary school 2) decreased target enrollment from 40 to 30 students for a lower student-to-trainer ratio (15 to 1, as opposed to 20 to 1). Of the 128 students in the study, a different number was present at baseline and follow-up measurements. Because this study evaluated participation in the program, we did not count the number of students who completed the program (Figure 2).

At baseline, 60 (55%) participating students met criteria for overweight, with a BMI in the 85th to the 95th percentile for age and sex, or obesity, with a BMI in the 95th percentile or higher for age and sex (28). These percentages were slightly higher than the district-wide prevalence of 45% of students with a BMI in the 85th percentile or higher (18). Parents provided specific responses for reasons to enroll their children in the BOKS program (Table 2).

**Table 2:** Summary of Relevant Dimensions and Representative Feedback From Parent Interviews, Build Our Kids’ Success (BOKS) Evaluation, Massachusetts, 2018^a^^,^ b

RE-AIM Dimension	Relevant Interview Question(s)	Key Themes and Subthemes	Representative Quotes
**Reach**	Your child has been participating in the BOKS program at their school; why did you choose to enroll them in this?	Benefits parent and child	• [I]t’s good because, for me, I have three kids. They study in two different schools. . . . When [teacher] said, “Now we have the BOKS program,” and also, “Sign up for the kids, and then they can come to school early.” (Father of daughter, 7 y) • I just said that maybe it’s gonna help him and put this energy a little down. (Mother of son 10, y)
		Child interest	• He loves anything that keeps him bouncing around and moving and jumping. He definitely was very interested in signing up, so, it was all him. (Mother of son, 9 y)
		Need for physical activity opportunities	• Oh, because I knew that at home he’s not very active and I will like to see him do more exercise and keep active, just to keep him healthy. (Mother of son, 6 y)
**Implementation**	What things have made it hard to participate in BOKS? Is there anything you would change about the program?	Acceptability	• From my standpoint, from my children, whatever is going on at BOKS, and you guys are doing, seems to be keeping my kids very interested. From my standpoint, I wouldn’t change a thing. (Mother of daughter, 9 y)
		Barriers: transportation, weather, time	• The first time it was hard because I’m not driving anymore. It was his father, and it was really hard for him, but now we did manage that. (Mother of son, 10 y) • [It’s hard] especially when it’s cold and raining. (Mother of daughter, 6 y) • Getting him ready and up early in the morning. The only downside. (Mother of son, 9 y)
		Suggestions for future program structure and content	• I think maybe a shorter program. I think that the hour was a very long time. (Mother of son, 9 y) • I wish that it’s more than 12 weeks, because my child likes that program very much. (Mother of son, 6 y) • It’s like, if they can do some dancing too in the morning, some music, some dance, some Zumba, something. (Mother of daughter, 6 y)
**Effectiveness**	What good things have you seen about participating in the BOKS program?	Impact on parents	• I found that it was helpful for me. . . . It helped me with the day. You know what I mean? (Mother of daughter, 6 y)
		Child benefits observed: behavior, self-esteem, health	• He concentrates better at school. His teacher’s not so after him to calm down. He’s got ADHD, so, I think it kinda helps him settle his mind a little bit having that activity in the morning so he’s not so wound up. (Mother of son, 9 y) • [S]he teach me how to do a new exercise. I don’t know how to do it but she teach me how to do it. . . . I like to see her more confident and active. (Mother of daughter, 8 y) • I see she’s losing a little weight. (Mother of daughter, 9 y)
		Physical activity behaviors: skills, enjoyment, sedentary time	• Coordination used to be a big deal with him, but he’s past that right now, so that’s why I think BOKS probably helped him. (Mother of son, 10 y) • They make it fun to be active. They play games. (Mother of son, 9 y) • [E]xercising to take her away from watching TV or to be[ing] inactive. (Mother of daughter, 10 y)

Abbreviations: RE-AIM, Reach, Effectiveness, Adoption, Implementation, and Maintenance.

a Based on Holtrop et al (29).

b Adoption not assessed as a setting-level domain; maintenance not assessed because interviews were performed during program (before maintenance period).

Demographics of consenting participants were representative of school and district demographics overall. Within the school district, 55.3% of students identify as Hispanic/Latino (participating schools range 43.5%–56.7%) and 34.3% of students identify as non-Hispanic white (participating schools range 33.3%–43.1%) (17). Most study participants identified as Hispanic/Latino (n = 52, 41%) on program enrollment forms, followed by non-Hispanic White (n = 29, 23%). (Table 3).Thirty (23%) of study participants returned parental consent forms in a language other than English. 

**Table 3:** Participant Demographics at Baseline by School and by Session in the Build Our Kids’ Success (BOKS) Evaluation, Massachusetts, 2018

Characteristics	Total (N = 128)^a^	By School				By Session		
		Elementary School 1 (n = 50)	Elementary School 2 (n = 47)	Middle School (n = 31)	*P* Value^b^	Spring (N = 86)	Fall (N = 42)	*P* Value^b^
Age, mean (SD), y	9.3 (2.2)	8.3 (1.6)	8.4 (1.6)	12.2 (0.7)	<.01	9.3 (2.2)	9.2 (2.2)	.90
Male, no. (%)	61 (48)	26 (52)	20 (43)	15 (48)	.57	45 (52)	16 (38)	.90
Baseline BMI, kg/m^2^, mean (SD)^a^	20.4 (4.4)	19.2 (2.5)	20.0 (4.2)	21.4 (4.4)	.05	20.3 (4.0)	20.5 (5.2)	.06
Baseline BMI *z* score, median (IQR)^c^	1.16 (0.39, 1.73)	1.24 (0.60, 1.82)	1.39 (0.44, 1.96)	0.64 (.21, 1.39)	.12	1.26 (0.40, 1.87)	1.32 (0.50, 1.55)	.47
**Child BMI Category, no. (%)**					.17^d^	—	—	.50^d^
<85th percentile, no. (%)	50 (45)	18 (43)	14 (35)	18 (64)	—	34 (45)	13 (38)	—
85th–95th percentile	27 (25)	17 (40)	16 (40)	6 (21)	—	17 (22)	10 (29)	—
>95th percentile	33 (30)	7 (17)	10 (25)	4 (14)	—	25 (33)	8 (24)	—
**Race/ethnicity, no. (%)**					—	—	—	.90
Hispanic/ Latino	52 (41)	26 (52)	15 (32)	11 (35)	—	31 (36)	21 (50)	—
Non-Hispanic White	29 (23)	10 (20)	11 (23)	8 (26)	—	20 (23)	9 (21)	—
Non-Hispanic Black	8 (6)	3 (6)	3 (6)	2 (6)	—	6 (7)	2 (5)	—
Other	13 (10)	6 (12)	6 (13)	1 (3)	—	9 (11)	4 (9)	—
Declined	26 (20)	5 (10)	12 (25)	9 (29)	—	20 (23)	6 (14)	—

Abbreviation: — , not applicable; BMI, body mass index.

a N = 112 total participants with complete baseline anthropometrics for BMI calculation, 78 in spring, 34 in fall.

b *t* tests, ANOVA, and Wilcoxon tests were used for continuous variables; χ^2^ tests used for categorical variables; race/ethnicity by school not assessed because of insufficient sample size.

c For BMI *z*-score calculation, total N = 110 participants, 2 students are missing data on age.

d *P* values represent χ^2 ^analysis for BMI category across schools and sessions.

### Implementation

#### Institutional support

Logistical support for programming was provided by the district superintendent, school principals, and the BOKS program. Each school managed program location, dates, and times, as well as student program enrollment and trainer recruitment.

#### Program costs

Total program cost for each 12-week session per school was $2,600 and included trainer stipends, gym equipment ($300 per school), and participant t-shirts (approximately $300 per school). The program curriculum was free of cost. Assuming full capacity, total cost per participant was $65/student; at actual capacity, cost was $83/student. Program costs for the study period were grant funded; therefore, participation was free for students and participating schools.

#### Program fidelity

**Structure.** All schools adhered to recommended program frequency (3 times/week) and scheduled program duration (12 weeks total). Schools completed 32 to 36 sessions, or 89%–100% of scheduled sessions. Reasons for missed sessions included inclement weather (n = 2) or a school event (n = 2). Recommended program time was 60 minutes; as implemented, sessions were 25 minutes at the middle school, 45 minutes at elementary school 1, and 60 minutes at elementary school 2. Both schools that were unable to achieve the prescribed length were limited by early morning access to school facilities and school start time. Overall program capacity was 40 students (1 trainer per 20 students).

**Content.** We performed 10 structured observations using the SOFIT observational measure. Interobserver reliability was 87% for activity observations and 84% for context observations. Across SOFIT-observed sessions, mean 19% (SD, 9) of sessions were spent in vigorous activity, 14% (SD, 7) standing, 37% (SD, 13) walking, 28% (SD, 16) sitting, and 3% (SD, 7) lying down. For lesson context, 54% (SD, 13) of the lesson was spent on fitness, skills, or games, 43% (SD, 12) was spent on management/knowledge, and 3% (SD, 5) was classified as other.

**Attendance.** Overall participant attendance was 77.2% (IQR, 17.5%–95.1%) of sessions, with significantly higher attendance among study participants (median = 90%; IQR, 56%–97%) versus program enrollees who did not participate in the study (median 23%; IQR, 9%–89%; *P* < .001). No significant difference in session attendance occurred from the first to the second program sessions among schools in 2018. Median attendance in spring 2018 was 91% (IQR, 63%–95%) of sessions. Median attendance in fall was 84% (IQR, 48%–97%) of sessions (*P* = .89) (Table 4).

**Table 4:** Program Attendance by School, Session, Student Identification as Hispanic/Latino, and Participation Status, Build Our Kids’ Success Evaluation, Massachusetts, 2018

Variable	No. of Sessions Attended, Median (IQR)	*P* Value^a^	Percentage of Sessions Attended, Median (IQR)	*P* Value^a^
**By school**				
Elementary school 1 (n = 50)	30 (24–31)	<.001	94 (75–97)	.04
Elementary school 2 (n = 31)	37 (29–40)		93 (69–95)	
Middle school (n = 47)	16 (8–21)		57 (35–91)	
**By session**				
Spring (n = 86)	29 (18–35)	.61	91 (63–95)	.89
Fall (n=42)	30 (15–31)		84 (48–97)	
**By study participation**				
Yes (n = 128)	29 (18–33)	.001	90 (56–97)	.001
No (n = 100)	7 (2–31)		23 (9–89)	
**By Hispanic/Latino**				
Yes (n = 52)	27 (1–31)	.38	87 (66–97)	.91
No (n = 76)	30 (15–36)		91 (48–97)	

a Wilcoxon rank sum used because of non-normality of data.

**Physical activity delivery**. Overall, 32% to 35% of the session was spent in MVPA, with no significant differences in percentage of time in MVPA between schools (Table 5). Total MVPA was moderately correlated with program duration (*r* = .36, *P* = .008). 

**Table 5:** Overall Time Spent in Moderate-to-Vigorous Physical Activity (MVPA) and Percentage of Participants Achieving Physical Activity Targets, Build Our Kids’ Success Evaluation, Massachusetts, 2018

Measure	Total (N = 84)	Elementary School 1 (N = 30)	Elementary School 2 (N = 39)	Middle School (N = 15)	*P* Value
Mean wear time, mean (SD), min	48.0 (11.6)	44.7 (0.4)	59.9 (0.28)	29.0 (1.3)	<.001^a^
MVPA, mean (SD), min	16.3 (9.3)	15.8 (6.0)	19.1 (11.6)	9.7 (2.0)	.003^a^
Percentage of sessions in MVPA, mean (SD)	33 (16.0)	35 (14.0)	32 (19.0)	33 (7.0)	.68
**Students achieving physical activity target, no. (%) **					
≥5 min of MVPA	84 (100.0)	30 (100.0)	39 (100.0)	15 (100.0)	—b
≥10 min of MVPA	64 (76.2)	23 (76.7)	36 (92.3)	5 (33.3)	—b
≥15 min of MVPA	32 (38)	14 (47)	18 (46)	0	—b
≥20 min of MVPA	21 (25)	10 (33)	11 (28)	0	—b
≥30 min of MVPA	6 (7)	0	6 (15)	0	—b

Abbreviation: NA, not applicable.

a One-way ANOVA used for continuous variables of minutes and percentage of time spent in MVPA.

b χ^2^ tests not performed because of insufficient sample size for categorical variables of student percentage meeting physical activity targets.

#### Feasibility and acceptability

Parent feedback on program implementation relating to feasibility, acceptability, barriers, and suggestions for future programming supported positive program outcomes. 

### Effectiveness

Effectiveness evaluation revealed maintenance in BMI (mean change −0.1 kg/m^2^, 95% CI, −1.2 to −1.0), BMI *z* score (mean change −0.001 units, 95% CI, −0.3 to 0.3) and self-reported quality of life (mean change in PedsQL total score of −0.8 units, 95% CI, −5.2 to 2.8) from baseline to completion of program at the 12-week follow-up. Parent observations regarding impact of program participation are summarized in Table 2.

### Maintenance

Enrollment decreased between sessions from 103 students (120 capacity) in spring 2018 to 85 (110 capacity) that fall. The decrease was primarily a result of decreased middle school enrollment. Both elementary schools had sustained interest, reaching 100% enrollment capacity in the second session (70 participants, including 67 new participants with 3 continuing siblings whose mother volunteered) with an additional 143 students on wait lists. In elementary school 1, 20 of 36 spring participants were on the fall wait list. Elementary school 2 solicited enrollment paperwork only from new participants. No middle school student participated in both sessions.

No significant difference in session attendance occurred from the first to the second program sessions among schools in 2018. Median attendance in spring 2018 was 91% (IQR, 63%–95%) of sessions. Median attendance in fall was 84% (IQR, 48%–97%) of sessions (*P* = .89) (Table 4).

In spring 2019, the program continued in schools with district-supported funding and expanded to 3 additional schools (2 elementary, 1 middle school) through additional grant funding for a total of 6 schools, representing 61% of the district’s K–8 enrollment. Total enrollment was 189 students of 230 for spring 2019 (82% capacity; 115 of 120 in new schools, 72 of 110 in previously participating schools). Overall, 17 trainers led the spring 2019 session (2 recruited within each new school; 8 continuing within previous schools, with 3 additional trained). Trainers who did not continue cited the time and administrative burden; elementary school 1 addressed this by splitting responsibilities among 3 trainers.

## Implications for Public Health

Using the RE-AIM framework to guide our implementation evaluation, we observed that each of the 3 participating schools successfully implemented the BOKS program. Each school had different approaches to program delivery, with potential implications for program results and sustainability. Overall, schools that adopted the program were representative of the school community at large, successfully reaching a diverse target population at risk for obesity-related sequelae. Program implementation varied most between schools in session length and structure of trainer teams. Both elementary schools had sustained interest between the spring and fall sessions, although interest decreased in the middle school. These findings should be considered in dissemination and delivery of school-based physical activity programs, as well as broader population health efforts to increase access to physical activity opportunities in diverse and low-resource settings.

In collaboration with stakeholders, our implementation strategy prioritized program adaptability within each school’s capabilities, maximizing fidelity through ongoing support and training from BOKS and school-based coordinators (30). Literature describing successfully disseminated physical activity interventions supports the importance of in-person, hands-on training, as well as building self-efficacy and ownership in the target community, both of which were priorities in our approach. Although counterintuitive, larger dissemination efforts show that omitting critical intervention components might be necessary for success (31). In low-resource settings, especially such as in this study, successful adaptation necessitates balancing complete fidelity with practical constraints.

Limited space and schedule constraints warranted decreased session time in 2 schools from the prescribed 60 minutes. With one-third of session time spent in MVPA, a further reduction influenced the total time promoting MVPA to participants. Fixed logistical constraints and competing priorities are common barriers to implementing wellness initiatives in schools and are not unique to this study (32). As a result, strategies to maximize MVPA within the available time are crucial. Our program observations suggest targets for improvement, mostly related to minimizing time spent in program management. Potential strategies include simplifying program activities to reduce the time spent explaining rules, directing children to move in place (eg, jogging, jumping jacks) during explanations, and promoting active cheering during activities that require taking turns (eg, relays).

Schools also adapted their approach to trainer recruitment, either concentrating responsibilities between 2 trainers or distributing responsibilities among a larger group. Program sustainability depends on maintaining trained personnel who are willing to deliver the intervention (31). With volunteer trainers who might have competing professional commitments, it is crucial to distribute the burden and build program champions while avoiding burn-out, especially in a low-resource school.

After the first year, stakeholder discussions focused on program sustainability. Additional grant funding supported program expansion; however, our shared priority was to identify existing funding streams in the district budget to support trainers. School wellness personnel and current trainers also proposed strategies requiring minimal funding, including a peer mentorship model with older student volunteers. These strategies rely on fitting the current intervention within existing school programs and policies as well as continuing to build capacity (33).

Ultimately, program results depend on overall reach and efficacy (31). At maximum capacity in the initial schools, the BOKS program could accommodate only 17% of students. As schools considered program continuation, they weighed having a different group of students participate each morning. When time and personnel are limited, increasing reach would have an unavoidable trade-off of decreasing overall MVPA delivery to each participant. The balance depends on individual priorities for program implementation. Although the research setting optimizes dose and program adherence, the community setting might prioritize equity in access. Identifying scalable and effective strategies to supplement school-based physical activity interventions is a research priority to extend reach and maintain the optimal intervention dose.

Although this intervention successfully engaged a diverse group of youth at risk for obesity-related sequelae, students not enrolled in the study had lower levels of program attendance. Although those students might have been less interested in the program, other disparities might exist between the study and nonstudy groups. Although we translated our study consent forms to reduce language barriers, both overall literacy and health literacy might have influenced enrollment. As vulnerable populations have lower rates of intervention delivery (34), social determinants of health or other unmeasured barriers to participation must be considered to account for differences in program participation.

Additionally, the middle school had high rates of attrition compared with both elementary schools. Although school leadership changed between sessions, an identified risk factor for poor sustainability (32), other factors unique to adolescents also warrant consideration. Adolescence is a time of increasing independence and physical and emotional changes (35). Lifestyle interventions that target overweight and obesity are often less successful in adolescents than in younger children (36). In our population, fewer middle school participants had BMI higher than the 85th percentile compared with elementary school participants. This lower prevalence of overweight and obesity suggests that middle school students most at risk for obesity-related sequelae were less likely to participate in the program than those with BMI <85th percentile. These factors suggest that the program might need to be tailored to improve uptake among adolescents.

Our study’s primary strength is its use of a structured framework, RE-AIM, to evaluate a before-school physical activity program. This approach provides a comprehensive view across each measure with qualitative insights from parents and a focus on identifying program adaptations needed for successful implementation. We identified key objectives for program improvement, which can be explored through more work on processes to maximize physical activity delivery. Furthermore, using the before-school period to deliver a physical activity intervention is an innovative approach, as it takes advantage of a time when children and adolescents are not typically active.

Although we have detailed information on implementation in each school, we were not able to test implementation strategies between schools. Without a greater number of participating schools, differences in each school’s capabilities would have confounded differences in implementation strategies. School district stakeholders identified participating schools, which might limit generalizability. Although schools were representative of the district, other unmeasured characteristics, such as openness to innovation or school leadership qualities, may have made these early adopters likely to succeed (10).

Additional perspectives are missing from this study, including teacher input, detailed trainer feedback, and qualitative interviews with non–English-speaking and middle school parents. For parent interviews, adequate representation would have required translation to Arabic, Portuguese, and Spanish, which was not feasible. Although we focused on parents of elementary students, challenges in the middle school setting highlight the importance of engaging the middle school population and provide a target for future work. This study was performed within a single school district and results may not be generalizable beyond this community; however, the evaluation process used may be applied across different school settings.

This study demonstrates that structured implementation and evaluation of an evidence-based physical activity program in a low-resource setting is feasible and yields relevant information on program delivery. Program adaptations may be crucial to successful implementation; however, they might also have implications for program outcomes. Through structured implementation evaluation following a similar procedure to this study, it may be possible to identify program components that are key to successful implementation, allowing for targeted dissemination and diffusion of the effective intervention components across various settings.
